# Gut Microbiome and Metabolome Modulation by High-Hydrostatic-Pressure-Processed Tomato Juice

**DOI:** 10.3390/nu16050710

**Published:** 2024-02-29

**Authors:** Xuehua Wang, Daotong Li, Chen Ma, Xiaosong Hu, Fang Chen

**Affiliations:** 1 College of Food Science and Nutritional Engineering, National Engineering Research Centre for Fruits and Vegetables Processing, Beijing Key Laboratory for Food Non-Thermal Processing, China Agricultural University, Beijing 100083, China; wangxuehua@whpu.edu.cn (X.W.); lidaotong@cau.edu.cn (D.L.); machen21@sina.com (C.M.); huxiaos@263.net (X.H.); 2 National R&D Center for Se-Rich Agricultural Products Processing, Hubei Engineering Research Center for Deep Processing of Green Se-Rich Agricultural Products, School of Modern Industry for Selenium Science and Engineering, Wuhan Polytechnic University, Wuhan 430023, China

**Keywords:** tomato juice, high hydrostatic pressure, gut microbiota, metabolic profiles, SCFAs

## Abstract

High hydrostatic pressure (HHP) is a non-thermal pasteurization technology for the enhancement of food products’ safety and quality. The components of tomato juice can be affected by HHP processing. Little is known about the effects of HHP-processed tomato juice on the gut microbiome and metabolism. Here, we performed high-throughput sequencing and metabolomics profiling to determine the critical differences in gut microbiota structure and metabolic profiles in mice administered with HHP-processed tomato juice. Tomato juice administration significantly increased the gut bacterial alpha diversity and the relative abundance of Bacteroides. The mice administered with HHP-processed tomato juice were characterized by the enrichment of Bacteroidetes, *Alistieps*, and *Faecalibaculum* compared with those administered with HTST-processed tomato juice. Moreover, HHP-processed tomato juice promoted SCFA levels, which were positively correlated with the enriched *Alistieps*. Our results show that HHP-processed tomato juice may drive healthy gut microbes and metabolites.

## 1. Introduction

The demand to make food healthier, safer, tastier, and more shelf stable promotes the development of innovative technology. As one of the most important non-thermal processing technologies, high hydrostatic pressure (HHP) can inactivate foodborne microorganisms and enzymes to extend food products’ shelf-life. Compared to conventional thermal processing, HHP processing can maintain the nutritional and sensory properties of fresh products due to the lower treatment temperature [1]. Therefore, HHP processing is commonly used to manufacture and preserve fruit- and vegetable-based products which maintain diverse bioactive compounds, flavors, and colors [2]. A study showed that HHP processing (600 MPa, 5 min) preserved greater levels of color, total phenolics, total flavonoid, and antioxidant properties of grapefruit juice than thermal treatment at 85 °C for 45 s [3]. Another study reported that the contents of volatile compounds (aldehydes and alcohols) were higher in mulberry juice subjected to HHP processing at 500 MPa for 10 min compared to that processed by thermal treatment [4]. In addition, a randomized cross-over clinical trial study revealed that the glycemic index was improved after consumption of HHP-processed mango puree [5]. HHP processing promotes the preservation of functional components and may potentially enhance the nutritional value of food products for human health. There is an urgent need to evaluate the health-promoting impacts of HHP-processed food products on human health.

The intestinal microbiota is a complex and diverse community of microbes that live in the gastrointestinal tract. Studies have confirmed that the gut microbiota plays an important role in host health. Dysbiosis of the gut microbiome has been implicated in the pathogenesis of various human diseases, for example, irritable bowel syndrome, obesity, and type 2 diabetes [6]. Diet profoundly alters the structure and function of intestinal microbiota [7,8,9], as dietary nutrients directly interact with the gut microbiota to promote or inhibit their growth. In return, the gut microbiota is also involved in the metabolism of dietary ingredients. The diet–microbiota interactions produce intestinal microbial metabolites, like short-chain fatty acids (SCFAs), which participate in various physiological functions related to intestinal homeostasis [10]. Thermal treatment alters the physicochemical properties of foods. Food processed by thermal treatment significantly reduced gut microbial diversity in catfish and mice [11]. In addition, intense roasting and grilling of bananas or bread can decrease levels of healthy bacteria [12]. Therefore, the structure and functionality of gut microbiota can be affected by food thermal processing. However, few studies have attempted to investigate the effects of HHP-processed food products on the gut microbiome and metabolome.

Tomatoes (*Lycopersicon esculentum*) are one of the world’s most productive agricultural products, with a global tomato yield of 186,821,216 tonnes in 2020 [13]. Tomato is an important source of nutrients and bioactive components such as dietary fiber, minerals, vitamins, proteins, and essential amino acids, which have extensive physiological properties, such as anti-inflammatory and antioxidant activities [14]. As the second most commonly consumed vegetable in the U.S., most tomatoes are consumed in the form of processed products, like ketchup, tomato powder, and tomato juice [14]. Supplementation of tomato power significantly up-regulated the abundance of Bacteroidetes and down-regulated the abundance of Firmicutes in DSS-induced colitis mice [15]. Additionally, an increased abundance of *Lactobacillus* and a decreased abundance of *Clostridium* were found in high-fat-diet mice administered with tomato juice [16]. Thus, consuming tomato products induces profound variation in the gut microbiome.

Our previous study has demonstrated that HHP-processed tomato juice has a distinct profiling of metabolites compared to that processed by high-temperature short-time processing (HTST). The contents of β-carotene, lycopene, quercetin, and ascorbic acid, and the relative levels of some aldehydes, alcohols, and amino acid derivatives, were found to be higher in tomato juice processed by HHP than by HTST [17]. Therefore, we postulate that the distinct metabolite profiles and chemical composition in HHP- and HTST-processed tomato juice may shape a different gut microbiome. This study focuses on (i) investigating the effect of tomato juice on the gut microbiota structure and metabolic profiles in mice; and (ii) identifying critical differences in gut microbiota structure and metabolic profiles of mice supplemented with HHP- and HTST-treated tomato juice. Our study provides new insights into the benefits of HHP processing of tomato juice from the perspective of improving the gut microenvironment and contributes a basis for the application of non-thermal technology.

## 2. Materials and Methods

### 2.1. Tomato Juice Preparation

Maturity “Heinz Series” tomatoes were harvested in Xinjiang Province and obtained in August 2019. The fruit samples were processed according to our previous study’s process to obtain HHP-treated (550 MPa/10 min), HTST-treated (110 °C/8.6 s), and fresh (untreated) tomato juice samples [17]. After processing, samples were instantly stored at −80 °C until given to mice.

### 2.2. Animal Study

C57BL/6J mice (9 weeks old) were housed in a 12 h light/dark cycle maintained under standard pathogen-free (SPF) conditions at 20–22 °C and 45 ± 5% humidity. Mice were randomly divided into four groups (n = 12): fresh tomato juice (Fresh group), HTST processed tomato juice (HTST group), HHP-processed tomato juice (HHP group), and sterile saline (Blank group) at a dose of 10 mL/kg by oral gavage twice daily after 1 week of acclimatization. After 4 weeks, mice were killed after 12 h of food deprivation. Serum samples were obtained from blood samples with centrifugation (3000 rpm, 4 °C, 15 min). Cecum content samples were collected in sterilized tubes and then stored at −80 °C.

Animal experiments were conducted under the approval number AW09211202-4-1 at the Laboratory Animal Centre of China Agricultural University (Beijing, China) based on national legislation and local guidelines.

### 2.3. Blood Biochemical Assay

Low-density lipoprotein, high-density lipoprotein, total cholesterol, total triglycerides, alanine aminotransferase, and aspartate aminotransferase in serum were determined by a biochemical analyzer (AU480, Japan Olympus Corporation, Tokyo, Japan).

### 2.4. Gut Microbiota Analysis

Total genome DNA was extracted using the CTAB/SDS method from the cecum content samples. Primers 341F (5′-CCTAYGGGRBGCASCAG-3′) and 806R (5′-GGACTACNNGGGTATCTAAT-3′) were used to amplify the V3–V4 hypervariable regions of the 16S rRNA genes, and PCR amplification products were sequenced on an Illumina NovaSeq platform (250 bp, paried) at Novogene Bio-Pharm Technology (Beijing, China). Uparse software (Uparse v7.0.1001, http://drive5.com/uparse/ (accessed on 15 May 2020)) and the Silva Database (http://www.arb-silva.de/ (accessed on 25 May 2020)) were used to analyze sequences and screen the representative sequence for each OTU, respectively. Alpha (α) diversity and beta (β) diversity were analyzed with QIIME (version 1.9.1). Sequence data with the Bioproject ID PRJNA919275 were deposited in the NCBI Sequence Read Archive.

### 2.5. Analysis of Short-Chain Fatty Acids (SCFAs) by GC

A sample of 20 mg lyophilized cecum contents was homogenized with distilled water (0.8 mL) and 50% H_2_SO_4_ (0.4 mL), then extracted with 1.5 mL diethyl ether. The mixture was vortexed (room temperature, 3 min) and centrifuged (3000× *g*, 5 min); anhydrous CaCl_2_ was added to remove residual water in the supernatant, filtered (0.22 µm); and 1 μL supernatant was injected into a 2010 plus GC System (SHIMADZU, Kyoto, Japan). For separating SCFAs, SH-Stabilwax-DA (30 m × 0.32 mm × 0.50 µm) capillary column (SHIMADZU, Japan) and flame ionization detector were used. The initial oven temperature was set at 80 °C for 1.5 min, increased to 240 °C (10 °C/min), then maintained for 20 min. The injector and detector temperatures were 260 °C.

### 2.6. Metabolomics Analysis

The metabolomics study was carried out by Majorbio Bio-Pharm Technology (Shanghai, China). The fecal sample (50 mg) was mixed with 400 µL extraction solution (methanol:water = 4:1 (*v*/*v*), containing internal standard), treated with a high-throughput tissue crusher at 50 Hz for 6 min (−10 °C) and then at 40 kHz for 30 min, placed at −20 °C for 30 min, and centrifugated at 13,000× *g* for 15 min (4 °C). The supernatant was collected for analysis.

Thermo UHPLC system Tandem time-of-flight mass spectrometry UPLC-Q Exactive HF-X system was used for metabolite analysis. Chromatographic conditions are described in Table S1, and the column was set at 40 °C. MS conditions: ion source heating temperature, 400 °C; mass range: 70–1050 *m*/*z*; normalized collision energy, 20–40–60 V. Data-Dependent Acquisition mode was applied to acquire data. Peak detection and raw data alignment were performed on Progenesis QI 2.3 (Nonlinear Dynamics, Waters, Milford, MA, USA). The Human Metabolome Database (HMDB) (http://www.hmdb.ca/ (accessed on 5 September 2020)) and METLIN database (https://metlin.scripps.edu/ (accessed on 10 September 2020)) were used to identify mass spectra of metabolic features. Majorbio Cloud Platform (https://cloud.majorbio.com (accessed on 20 September 2020)) was used for subsequent analysis.

### 2.7. Statistical Analysis

The Kruskal–Wallis test was performed to compare medians between nonnormally distributed groups. The results from the experiments were calculated by a one-way ANOVA analysis. We used GraphPad Prism7.0 for graphing. For multivariate analysis, Principal Component Analysis (PCA) and Orthogonal Least-Partial-Squares Discriminant Analysis (OPLS-DA) were carried out by the R software package ropls (version 1.6.2). One-way PERMANOVA analysis was calculated by Past 4.02 (http://folk.uio.no/ohammer/past (accessed on 10 November 2020)). Variable importance in the projection (VIP) ≥ 1 and *p* < 0.05 were set as screening criteria for differential metabolite screening. Kyoto Encyclopedia of Genes and Genomes (KEGG, http://www.genome.jp/kegg/ (accessed on 25 September 2020)) was applied to analyze metabolic enrichment and pathways. Interventionary studies involving animals or humans, and other studies that require ethical approval, must list the authority that provided approval and the corresponding ethical approval code.

## 3. Results

### 3.1. Effect of Tomato Juice on Biochemical Parameters

There was no significant difference between mice administered with fresh, HHP-, and HTST-treated tomato juice in body weight, food consumption, and water intake (Figure S1). Consistently, there was no significant difference in the serum parameters, including low-density lipoprotein, high-density lipoprotein, total cholesterol, total triglycerides, alanine aminotransferase, and aspartate aminotransferase, between the treatment groups (Table S2).

### 3.2. Effect of Tomato Juice on Gut Microbiota Composition

To investigate whether administration with fresh, HHP-, and HTST-treated tomato juice altered the composition of the gut microbiota in mice, 16S rRNA gene sequencing was used. A total of 4,171,550 raw reads were picked up from 48 samples, averaging 86,907 ± 9121 reads per sample. A total of 1943 operational taxonomic units (OTUs) were determined (97% similarity) after quality checks, with an average of 431 ± 77 OTUs per sample.

**Figure 1:**
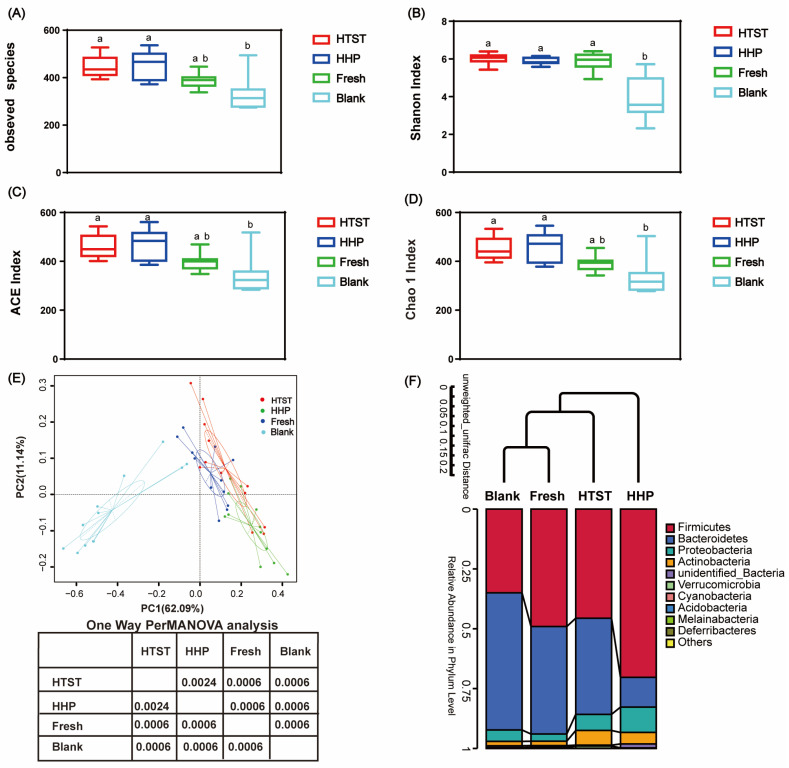
The diversity analysis of gut microbiota in mice administered with tomato juice. (**A**) Observed species; (**B**) Shannon index; (**C**) ACE index; (**D**) Chao1 index; (**E**) PCoA and one-way PERMANOVA analysis based on Weighted Unifrac; (**F**) UPGMA cluster tree analysis based on Unweighted Unifrac (n = 12). Blank: normal saline control group; Fresh: fresh tomato juice administration group; HHP: high-hydrostatic-pressure-processed tomato juice administration group; HTST: high-temperature-short-time-processed tomato juice administration group. Lowercase with different letters indicate significant differences (*p* < 0.05).

The bacterial alpha diversity refers to the bacterial species (OTUs) richness and evenness within a given region or ecosystem. The Chao index, ACE index, and observed species are measured to reflect species richness, while the Shannon index is used to reflect community evenness. As shown in Figure 1, the Shannon index was significantly higher in the three tomato juice administration groups than in the control group, suggesting that tomato juice administration increased the microbial community evenness in the mice. Interestingly, the observed species, ACE index, and Chao1 index were enhanced in mice administered with HHP- and HTST-treated tomato juice compared to the mice administered with fresh tomato juice, while there was no significant difference in the bacterial α diversity between the HHP- and HTST-treated groups (Figure 1).

PCoA based on OTUs showed that the groups administered with fresh, HHP-, and HTST-treated tomato juice displayed a similar clustering of the gut bacterial community relative to the control group. Notably, the PERMANOVA test showed a significant difference in the gut bacterial structure of these tomato juice treatment groups (*p* < 0.01) (Figure 1E). UPGMA clustering tree analysis revealed a significant difference in the bacterial β diversity between the HHP- and HTST-processed tomato juice groups (Figure 1F). Thus, these data suggest that administration of tomato juice, especially the tomato juice processed by HTST and HHP, significantly changed the structure of the intestinal microbial community in the mice.

**Figure 2:**
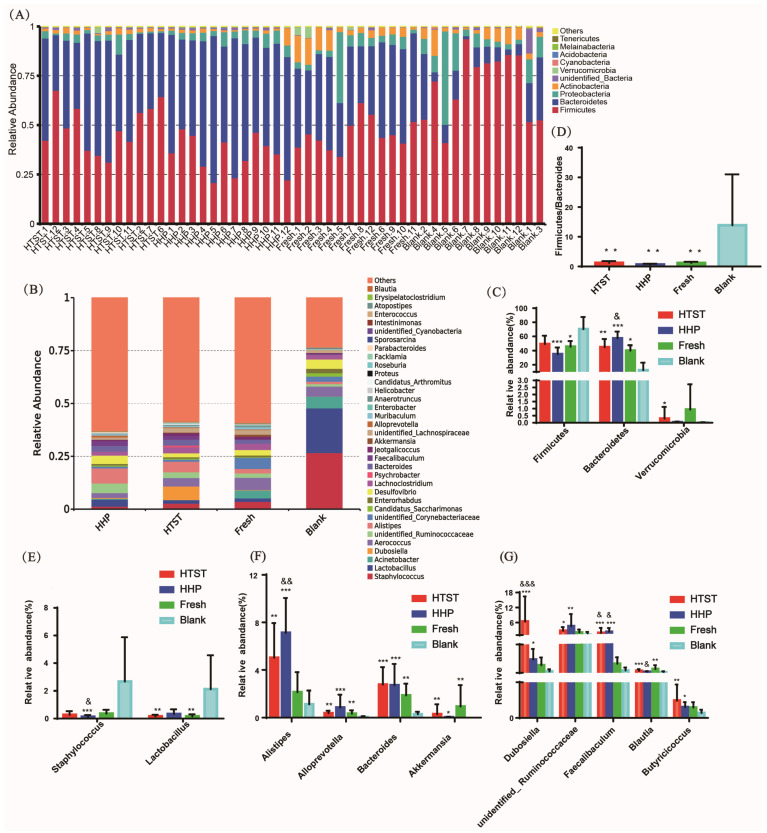
Tomato juice induces distinct gut microbiota composition. The gut bacterial composition at the (**A**) phylum level and (**B**) genus level; (**C**) the relative abundance of Firmicutes, Bacteroidetes, and Verrucomicrobia; (**D**) proportion of the Firmicutes vs. Bacteroidetes; the relative abundance of (**E**) *Staphylococcus* and *Lactobacillus*; (**F**) *Dubosiella*, unidentified-*Ruminococcaceae*, *Faecalibaculum*, *Blautia*, and *Butyricicoccus*; (**G**) *Alistipes*, *Alloprevotella*, *Bacteroides*, and *Akkermansia*. * and &: *p* < 0.05, ** and &&: *p* < 0.01, *** and &&&: *p* < 0.001; * indicates comparison with Blank group, & indicates comparison with Fresh group.

At different taxonomic levels, the bacterial communities and their relative abundance were further investigated. A total of 402 genera and 30 phyla were identified by gut bacterial taxa analysis. In tomato juice administration groups, the abundance of Firmicutes declined (Fresh: 45.62%, HHP: 35.00%, HTST: 49.08%, and Blank: 70.30%), whereas the abundances of Bacteroidetes (Fresh: 40.13%, HHP: 57.27%, HTST: 44.88%, and Blank: 12.43%) and Verrucomicrobia (Fresh: 0.94%, HHP: 0.04%, HTST: 0.29%, and Blank: 0.01%) were increased compared to the control group (Figure 2A,C). In addition, a lower ratio of Firmicutes to Bacteroidetes (F/B value) was found in the tomato juice groups compared to the control group (Figure 2D). At the genus level, the abundances of *Staphylococcus* (Fresh: 3.58%, HHP: 1.30%, HTST: 2.73%, and Blank: 26.68%) and *Lactobacillus* (Fresh: 1.64%, HHP: 3.33%, HTST: 1.64%, and Blank: 21.14%) decreased in tomato juice administration groups in comparison with in the control group (Figure 2B,E), while the abundances of *Dubosiella* (Fresh: 0.22%, HHP: 0.38%, HTST: 6.44%, and Blank: 0.05%), *unidentified-Ruminococcaceae* (Fresh: 2.06%, HHP: 4.57%, HTST: 2.72%, and Blank: 1.12%), *Faecalibaculum* (Fresh: 0.26%, HHP: 2.33%, HTST: 1.91%, and Blank: 0.08%), *Blautia* (Fresh: 0.10%, HHP: 0.04%, HTST: 0.07%, and Blank: 0.03%), *Butyricicoccus* (Fresh: 0.02%, HHP: 0.02%, HTST: 0.04%, and Blank: 0.01%), *Alistipes* (Fresh: 2.15%, HHP: 7.13%, HTST: 5.03%, and Blank: 1.12%), *Alloprevotella* (Fresh: 0.39%, HHP: 0.85%, HTST: 0.38%, and Blank: 0.28%), *Bacteroides* (Fresh: 1.87%, HHP: 2.72%, HTST: 2.77%, and Blank: 0.28%), and *Akkermansia* (Fresh: 0.94%, HHP: 0.03%, HTST: 0.29%, and Blank: 0.002%) increased in tomato juice administration groups compared to the control group (Figure 2B,F,G).

The linear discriminant analysis (LDA) effect size (LEfSe) algorithm was applied to identify the specific microbial taxa that were enriched in mice receiving different processed tomato juice administration (Figure S2). Our results showed that fresh tomato juice was associated with the enrichment of Bacteroidetes, *Muribaculaceae*, *Clostridia*, *Ruminococcaceae*, *Lachnospiraceae*, and *Corynebacterium_glutamicum* (Figure S2A). The HHP-processed tomato juice was associated with the enrichment of Bacteroidetes, *Muribaculaceae*, *Clostridia*, *Ruminococcaceae*, *Alistieps*, *Prevotellaceae*, *Bacteroides*, and *Faecalibaculum* (Figure S2B). The HTST-processed tomato juice was associated with the enrichment of Bacteroidetes, *Muribaculaceae*, *Clostridia*, *Ruminococcaceae*, *Lachnospiraceae*, *Alisteps*, *Bacteroides*, and *Dubosiella* (Figure S2C). Bacteroidetes, *Muribaculaceae*, *Clostridia*, and *Ruminococcaceae* were the bacteria commonly enriched in the three tomato juice groups.

### 3.3. Effect of Tomato Juice on Metabolic Profiles

Accordingly, we performed the metabonomics to analyze the metabolic profiling of fecal samples from mice administered with fresh, HHP-, and HTST-treated tomato juice. A total of 936 metabolites were identified after extraction, interpretation, and analysis of the raw spectral data. Based on the category of the KEGG library, the identified metabolites included the classes of amino acids (14), monosaccharides (8), vitamins (5), and fatty acids (6) (Figure S3A). Based on the category of the HMDB library, the metabolites mainly included 239 lipids and lipid-like molecules (41.42%), 115 organic acids and derivatives (19.93%), 74 organoheterocyclic compounds (12.84%), 36 benzenoids (6.24%), 35 phenylpropanoids and polyketides (6.07%), 35 organic oxygen compounds (6.07%), 22 organic nitrogen compounds (3.81%), and 16 nucleosides, nucleotides, and analogues (2.77%) (Figure S3B).

**Figure 3:**
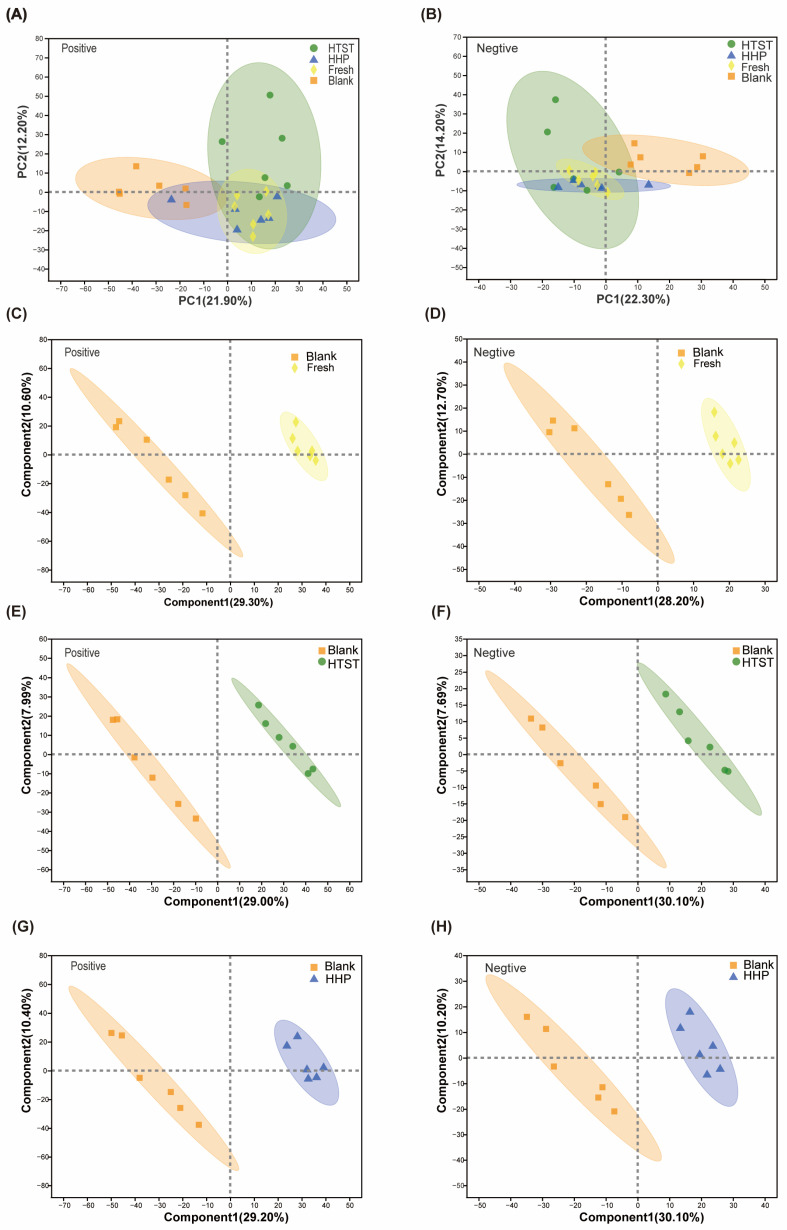
Tomato juice induces different fecal metabolomic profiles. PCoA plots are based on (**A**) anions and (**B**) cations cluster analysis of metabolites among groups (PLS-DA score plots). (**C**) Fresh vs. Blank, positive ion mode (R2X = 0.399, R2Y = 0.993, Q2 = 0.859); (**D**) Fresh vs. Blank, negative ion mode (R2X = 0.498, R2Y = 0.999, Q2 = 0.909); (**E**) HTST vs. Blank, positive ion mode (R2X = 0.37, R2Y = 0.997, Q2 = 0.835); (**F**) HTST vs. Blank, negative ion mode (R2X = 0.378, R2Y = 0.993, Q2 = 0.783); (**G**) HHP vs. Blank, positive ion mode (R2X = 0.397, R2Y = 0.99, Q2 = 0.877); (**H**) HHP vs. Blank, negative ion mode (R2X = 0.403, R2Y = 0.978, Q2 = 0.806).

PCA analysis showed that there was an apparent clustering of metabolic profiles in mice administered with the fresh, HHP-treated, and HTST-treated tomato juice in comparison with the control mice, suggesting that tomato juice administration altered the fecal metabolome in the mice (Figure 3A,B). Although no significant difference in metabolic profiles was found between the HHP- and HTST-treated tomato juice groups, PLS-DA analysis revealed dramatic metabolic differences between each tomato juice treatment group and the control group (Figure 3C–H).

**Figure 4:**
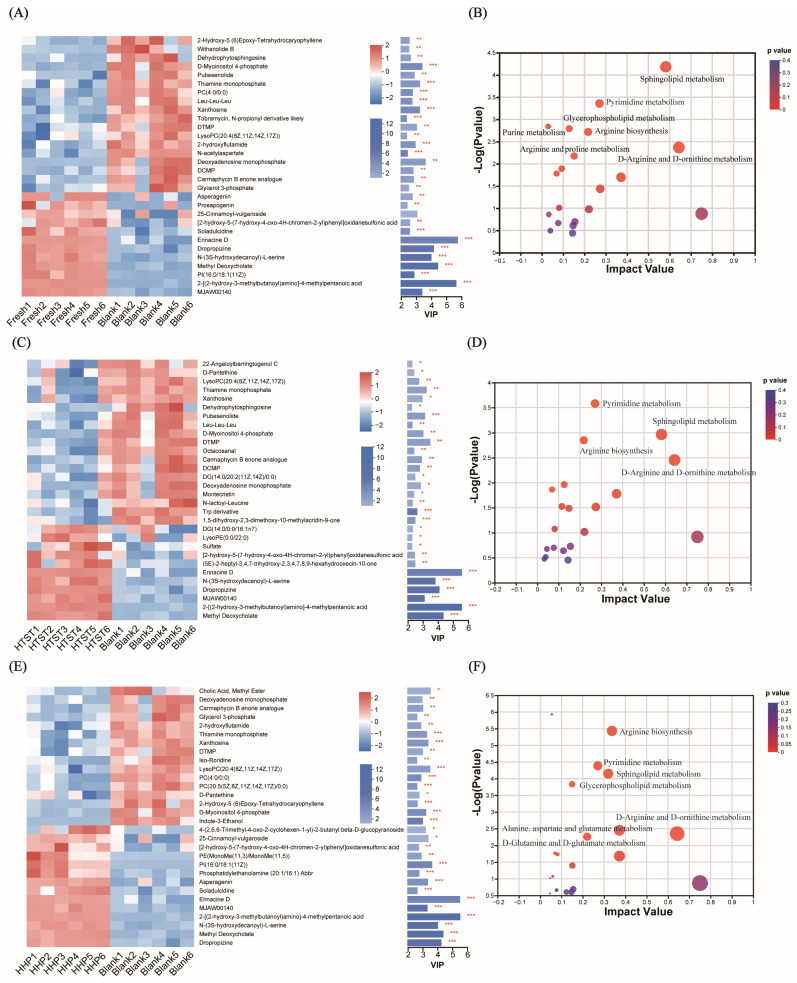
Differential metabolite analysis based on the top-30 expression profile and functional enrichment analysis of KEGG topology analyses; bubble graphs show comparison between control group and the (**A**,**B**) fresh, (**C**,**D**) HTST-processed, (**E**,**F**) HHP-processed tomato juice groups (* *p* < 0.05, ** *p* < 0.01, *** *p* < 0.001). The *Y* axis is the impact value of the relative importance of metabolites in the pathway, and the *X* axis is the enrichment of significant metabolites involved in the pathway −log10 (*p*-value). The bubble represents a KEGG pathway, and its size indicates the impact value. The text with the pathway name represents the significant corrected *p*-value (<0.05).

A total of 207 significantly altered metabolites were identified between the fresh tomato juice group and the control group (Table S3). The top-30 expression profile and VIP of metabolites in fresh tomato juice compared with control groups are shown in Figure 4A. Metabolites that significantly declined in the fresh tomato juice group included dehydrophytosphingosine, LysoPC(20:4(8Z,11Z,14Z,17Z)), d-myoinisitol-4-phosphate, thiamine monophosphate, PC(4:0/0:0), Leu-Leu-Leu, *N*-acetylaspartate, and glycerol 3-phosphate. In contrast, those that were significantly increased included asperagenin, 25-cinnamoyl-vulgaroside, soladulcidine, erinacine D, dropropizine, n-(3s-hydroxydecanoyl)-l-serine, methyl deoxycholate, and PI(16:0/18:1(11Z)). The main differential metabolic pathways were sphingolipid metabolism, glycerophospholipid metabolism, arginine biosynthesis, arginine and proline metabolism, l-arginine and d-ornithine metabolism, purine metabolism, and pyrimidine metabolism (Figure 4B).

A total of 183 significantly altered metabolites were identified between the HTST-processed tomato juice group and the control group (Table S3). The top-30 expression profile and VIP of metabolites in HTST-processed tomato juice in comparison with control groups are shown in Figure 4C. Metabolites that significantly declined in the HTST-processed tomato juice group included Leu-Leu-Leu, *N*-lactoyl-Leucine, trp derivative, LysoPC(20:4(8Z,11Z,14Z,17Z)), dehydrophytosphingosine, xanthosine, DTMP, DCMP, DG(14:0/20:2(11Z,14Z)/0:0), and deoxyadenosine monophosphate. In contrast, those that were significantly increased included DG(14:0/0:0/16:1n7), LysoPE(0:0/22:0), sulfate, erinacine D, *N*-(3S-hydroxydecanoyl)-l-serine, dropropizine, 2-[(2-hydroxy-3-methylbutanoyl)amino]-4-methylpentanoic acid, and methyl deoxycholate. The essential differential metabolic pathways were sphingolipid metabolism, arginine biosynthesis, d-arginine and d-ornithine metabolism, and pyrimidine metabolism (Figure 4D).

A total of 200 significantly altered metabolites were identified between the HHP-processed tomato juice and control groups (Table S3). The top-30 expression profile and VIP of metabolites in the HHP-processed tomato juice compared with control groups are shown in Figure 4E. Metabolites that significantly declined in the HHP-processed tomato juice group included cholic acid methyl ester, deoxyadenosine monophosphate, glycerol-3-phosphate, thiamine monophosphate, PC(4:0/0:0), xanthosine, LysoPC(20:4(8Z,11Z,14Z,17Z)), DTMP, indole-3-ethanol, and PC(20:5(5Z,8Z,11Z,14Z,17Z)/0:0), whereas those that were significantly increased included 25-cinnamoyl-vulgaroside, PE(MonoMe(11,3)/MonoMe(11,5)), PI(16:0/18:1(11Z)), phosphatidylethanolamine (20:1/16:1), asperagenin, soladulcidine, erinacine D, 2-[(2-hydroxy-3-methylbutanoyl)amino]-4-methylpentanoic acid, *N*-(3S-hydroxydecanoyl)-l-serine, methyl deoxycholate, and dropropizine. The main differential metabolic pathways were alanine, aspartate, and glutamate metabolism, arginine biosynthesis, d-arginine and d-ornithine metabolism, d-glutamine and d-glutamate metabolism, sphingolipid metabolism, pyrimidine metabolism, and glycerophospholipid metabolism (Figure 4F).

**Figure 5:**
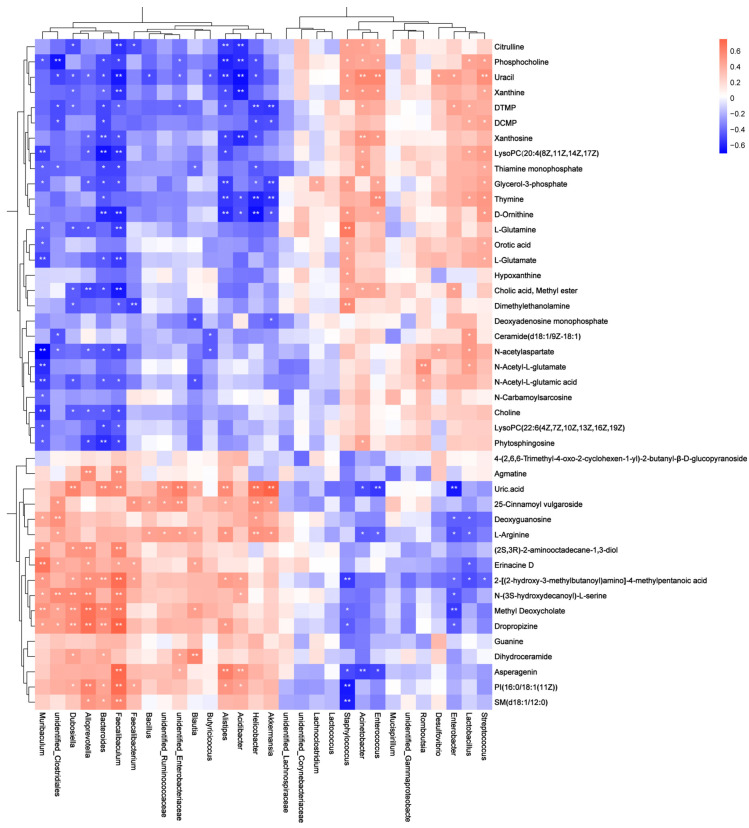
The Spearman correlation heatmap analysis between feature metabolites and gut bacteria. A significant correlation is indicated by an asterisk symbol (* *p* ≤ 0.05, ** *p* ≤ 0.01).

Correlation between significant genera relative abundance and feature fecal metabolites was studied using Spearman’s correlation coefficient. *Unidentified_Clostridiales* was positively correlated with *N*-(3S-hydroxydecanoyl)-l-serine and l-Arginine and was negatively correlated with *N*-acetylaspartate and *N*-Acetyl-l-glutamate (Figure 5). *Alloprevotella* was positively correlated with SM(d18:1/12:0) and PI(16:0/18:1(11Z)) and was negatively correlated with choline, phytosphingosine, and glycerol-3-phosphate. *Bacteroides* was positively correlated with PI(16:0/18:1(11Z)), dihydroceramid, methyl deoxycholate, 2-[(2-hydroxy-3-methylbutanoyl)amino]-4-methylpentanoic acid, and SM(d18:1/12:0) and was negatively correlated with choline, glycerol-3-phosphate, LysoPC(22:6(4Z,7Z,10Z,13Z,16Z,19Z)), cholic acid methyl ester, phosphocholine, and LysoPC (20:4(8Z,11Z,14Z,17Z)). *Faecalibaculum* was positively correlated with *N*-(3S-hydroxydecanoyl)-l-serine, SM(d18:1/12:0), and PI(16:0/18:1(11Z)) and was negatively correlated with *N*-acetylaspartate, d-ornithine l-glutamine, l-glutamate, *N*-Acetyl-l-glutamic acid, LysoPC(20:4(8Z,11Z,14Z,17Z), choline, phytosphingosine, phosphocholine, glycerol-3-phosphate, and LysoPC(22:6(4Z,7Z,10Z,13Z,16Z,19Z).

**Figure 6:**
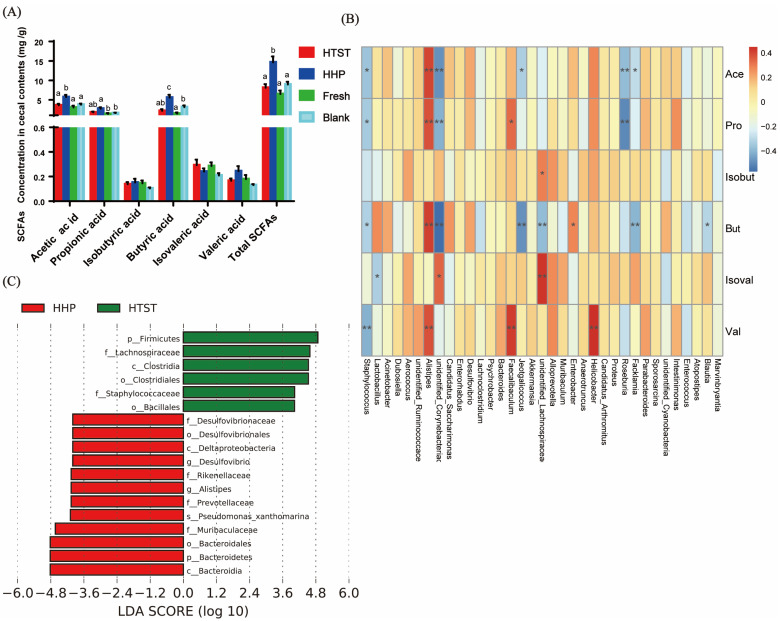
HHP-processed tomato juice increases SCFA concentrations and relative abundance of specific bacteria. (**A**) Acetic acid, propionic acid, isobutyric acid, butyric acid, isovaleric acid, valeric acid, and total short-chain fatty acid content; (**B**) correlation diagram of SCFAs and gut bacteria; (**C**) linear discriminant analysis effect size analysis to identify taxonomic differences in the gut microbiota of the mice administered with HHP- and HTST-treated tomato juice. Different letters indicate statistical differences between groups (* *p* ≤ 0.05, ** *p* ≤ 0.01).

The levels of SCFAs in feces were measured to evaluate the effect of HHP- and HTST-processed tomato juice on intestinal homeostasis. Our results demonstrated that the concentration of acetic, propionic, butyric acids, and total SCFAs was higher in the HHP-treated tomato juice group than in the control and other tomato juice groups (Figure 6A). Spearman rank correlation analyses further revealed the relationship between alterations in the abundance of different bacterial genera and the content of SCFAs (Figure 6B). It is noteworthy that the contents of acetic acid, butyric acid, and propionic acid were significantly and positively correlated with the abundance of *Alistieps*, which was bacterially enriched in mice administered with HHP-processed tomato juice. In contrast, the contents of these SCFAs were negatively associated with the abundance of *Staphylococcus*, which was enriched in mice administered with HTST-processed tomato juice (Figure 6C). Thus, the increased abundance of *Alistieps* and reduced abundance of *Staphylococcus* may have contributed to the higher SCFA levels in the HHP-treated tomato juice administration group compared to the HTST-processed tomato juice administration group.

## 4. Discussion

The beneficial impact of HHP processing on fruit juice has long been described, such as retention of better sensory and nutritional compounds than conventional thermal processing [2]. Studies have shown that the intestinal microbiome plays a significant role in host health. However, no study has compared the effects of HHP-treated and thermal-treated tomato juice on the gut microbiome and metabolome. This study demonstrated that the administration of tomato juice affects the structure of gut microbiota and metabolic profiles in mice. HHP-processed tomato juice promoted the enrichment of *Alistieps* and SCFAs, which can contribute to the physiological activities of host health.

According to the World Health Organization, a daily intake of 400–600 g of fruits and vegetables is recommended to decrease the risk of noncommunicable diseases and prevent malnutrition. Moderate fruit juice consumption (75–224 mL/d) is consistent with dietary health guidelines for the U.S. and several European countries [18]. In this study, 150 mL/d of tomato juice intake was selected as the human dose (60 kg) to convert into the mice equivalent dose (20 mL/kg/d) based on body surface area. A previous study also observed that replacing drinking water with tomato juice did not affect weight, food and water intake, or ALT and AST activities in the liver of SD rats [16]. Our results also showed that 4 weeks of tomato juice administration (20 mL/kg/d) had no effects on the body weight and serum parameters of mice. Thus, the human equivalent dose of tomato juice at a dose of 150 mL per day for adults could be recommended as no-weight-gain energy intake.

Diet is a major factor influencing host gut microbiota [7]. A previous study found that two weeks of tomato powder administration significantly increased the number of OTUs and the Shannon index [15]. Moreover, a decreased F/B value was observed in colitis mice with tomato powder supplementation [19]. It was reported that obese animals and humans had a higher abundance of Firmicutes and a lower abundance of Bacteroidetes, and the F/B value normalized with concomitant weight loss following a calorie-restricted diet [20]. In addition, Krajmalnik-Brown et al. [21] proposed that Firmicutes are more efficient at extracting energy from food than Bacteroidetes, making them more efficient at absorption. Tomato juice changed the structure of gut microbiota in mice by increasing the bacterial alpha diversity and decreasing the F/B value observed in our study. Our results suggest that tomato juice may be developed as a promising dietary supplement for the prevention of obesity through the reduction of the F/B value.

The relative abundance of Staphylococcus decreased, and the abundance of *Faecalibaculum*, *Alistipes*, *Alloprevotella*, *Bacteroides*, *Dubosiella*, unidentified-*Ruminococcaceae*, *Blautia*, *Butyricicoccus*, and *Akkermansia* increased in tomato juice administration groups compared to in the control group. In addition, *Ruminococcaceae* were the bacteria commonly enriched in the three tomato juice groups. Staphylococcus aureus is the primary pathogen that causes human clinical infection, while Staphylococcus epidermidis occasionally causes diseases. *Faecalibaculum rodentium* could slow down tumor growth by producing short-chain fatty acids [22]. *Alloprevotella*, *Bacteroides*, *Ruminococcaceae*, *Blautia*, *Butyricicoccus*, and *Akkermansia* are producers of SCFAs [23,24,25,26,27,28]. *Alistipes* was correlated with prolonged skin graft survival in mice [29], and high-fat diet-induced intestinal microbiota dysbiosis was reversed by the increased abundance of *Alloprevotella* [30]. *Bacteroides distasonis*, *Bacteroides uniformis*, and *Bacteroides ovatus* are involved in the hydrolysis of glycosidic bonds of flavonols (kaempferol, quercetin, etc.) [23]. Blautia is involved in the deglycosylation of polyphenols and the catabolism of lignans; its metabolites (such as butyric acid) contribute to the relief of inflammatory and metabolic diseases [25]. The increase in the relative abundance of *Dubosella* helps to prevent the development of salt-sensitive hypertension [31], and the abundance of *Ruminococcaceae* was significantly negatively correlated with the occurrence of alcoholic liver cirrhosis, hepatic encephalopathy, and non-alcoholic fatty liver disease [26]. *Butyricicoccus* supplementation alleviated colitis in rats, and the supernatant of butyric acid bacteria culture enhanced intestinal epithelial barrier function [27]. *Akkermansia muciniphila* is an intestinal symbiotic bacterium that colonizes the mucosal layer and has a high value in improving host metabolic function and immune response, as well as altering cancer treatment [28]. Previous studies found that tomato pomace decreased the abundance of Escherichia coli in vitro [32], and lycopene increased the bacterial alpha diversity and reduced the abundance of Clostridium in mice on a high-fat diet [33]. Moreover, tomato juice increased mice fecal concentrations of food-derived phenolics, which are essential in modifying the gut microbiota [16]. These results indicated that lycopene, dietary fiber, and polyphenols might contribute to the impact of tomato juice on gut bacterial community structure.

Diet regulates the structure of the gut microbial community. In turn, the gut microorganisms metabolize dietary components not utilized by the host, thereby influencing the co-metabolism of the host and its gut microbiota [8]. Metabolites, including *N*-acetyl-l-glutamate, (2S,3R)-2-aminooctadecane-1,3-diol, *N*-acetyl-l-glutamic acid, dihydroceramide, l-glutamate, l-arginine, and d-ornithine, were the common differential metabolites of the three tomato juice intervention groups. Notably, these metabolites were involved in sphingolipid metabolism, arginine biosynthesis, d-arginine, and d-ornithine metabolism. It has been reported that sphingolipids can be obtained from the diet by de novo synthesis in mammalian tissues. Moreover, the metabolism of gut bacteria Bacteroidetes is also considered an essential source of sphingolipids. *Bacteroides* and *Prevotella*, which possess serine palmitoyltransferase, are the Bacteroidetes that can synthesize sphingolipids from free sphingosine [34]. A significantly increased ceramide level was found in the liver of insulin-resistant mice supplemented with *Bacteroides thetaiotaomicron* [34]. Moreover, Bacteroides-derived sphingolipid levels were lower in IBD patients than in the average population. Bacteroides-derived sphingolipids, such as ceramide phosphatidol and deoxysphingolipids, were negatively correlated with inflammation and were influential in the maintenance of intestinal homeostasis and symbiotics [35]. Another study demonstrated that glycerophospholipids were broken down by phospholipases to generate arachidonic acid, while sphingomyelinases could regulate arachidonic acid to produce ceramide and phosphorylcholine [36]. The relative abundance of *Bacteroides* was positively correlated with PI(16:0/18:1(11Z)), dihydroceramid, and SM(d18:1/12:0), and negatively correlated with glycerol-3-phosphate, LysoPC(22:6(4Z,7Z,10Z,13Z,16Z,19Z), phytosphingosine, and LysoPC(20:4(8Z,11Z,14Z,17Z), in our study. Thus, the significant increase in *Bacteroides* may have contributed to the enrichment of sphingolipid metabolism in mice after tomato juice intervention. Previous studies indicated that *Clostridia* is the most common species involved in the fermentation of amino acids [37], and *Corynebacterium glutamicum* is a glutamate producer [38]. The reasons for the enriched arginine biosynthesis and d-arginine after tomato juice administration might be related to the rise of *Unidentified_Clostridiales*, and the d-ornithine metabolism change might be related to the enriched *Corynebacterium glutamicum*.

A primary finding of our study was that HHP- and HTST-processed tomato juice resulted in different gut microbiota structures and metabolic profiles of mice. HHP-processed tomato juice administration enhanced the relative abundance of *Bacteroides* and *Alistipes* and promoted SCFA production compared with HTST-processed tomato juice administration. SCFAs including acetic, lactic, propionic, n-butyric, i-butyric, n-valeric, and i-valeric acids are the essential fermentation products of gut microbiota that have a potential beneficial effect on human health [39]. Gomez et al. [40] showed that supplementation with high-dose lycopene (12 mg/100 g) tomato juice had no significant effect on the level of SCFAs in the intestinal system of rats, but low-dose (2.7 mg/100 g) lycopene increased butyrate levels. Our previous study observed that the concentration of total lycopene in HHP-processed tomato juice (7.98 mg/100 g) was higher than in HTST-processed juices (5.45 mg/100 g) [17]. Thus, the increased production of SCFAs in the HHP-processed tomato juice group might be related to the elevated concentration of lycopene content in tomato juice following HHP processing. Additionally, it has been reported that insoluble dietary fiber extracted from carrot pomace and mango pulp was modified by HHP [41]. Microbial fermentation can be affected by dietary fibers’ chemical composition and physicochemical properties [42]. All types of pectins could increase the level of acetic acid, but butyric acid was only enriched by pectin L13 [43]. Hence, the dietary fiber in tomato juice modified by HHP treatment was another possible reason for increased SCFA production. Notably, the levels of acetic acid, butyric acid, and propionic acid were significantly positively correlated with the relative abundance of *Alistieps*, which have been reported to produce acetic acids and iso-valeric acids [44]. It has also been reported that *Alistipes* has a beneficial role in cancer immunotherapy [29]. *Staphylococcus* is a potentially pathogenic bacterium, and studies found that *Staphylococcus* increased in the gut of inflammatory bowel disease and chronic rheumatic disease patients compared with ordinary people [45]. SCFAs can decrease *Staphylococcus aureus* internalization in the mammary glands [46]. Thus, the higher abundance of *Alistipes* might have promoted the accumulation of SCFAs, which, in turn, led to the lower abundance of *Staphylococcus* in mice administered with HHP-processed tomato juice. However, these hypotheses still need to be proven by further experiments, and the specific mechanisms that regulate the structure of the gut microbiota in mice treated with HHP-treated tomato juice still need to be further explored.

## 5. Conclusions

Our study demonstrated that tomato juice administration increased the gut microbiota α diversity and relative abundance of Bacteroides and regulated sphingolipid metabolism and arginine biosynthesis in mice. Moreover, we found that HHP-processed tomato juice increased the abundance of *Alistipes* and promoted the production of SCFAs compared with HTST-processed tomato juice. Our results provide a new insight into the advantage of HHP over HTST processing. This will benefit the application of non-thermal processing technology for the improvement of the gut microenvironment.

## Data Availability

16s RNA sequence data with the Bioproject ID PRJNA919275 were deposited in the NCBI Sequence Read Archive.

## Supplementary Materials

The following supporting information can be downloaded at: https://www.mdpi.com/article/10.3390/nu16050710/s1, Figure S1: (A) The body weight, (B) food consumption, and (C) water intake of mice administered with tomato juice.; Figure S2: Linear discriminant analysis effect size analysis to identify taxonomic differences in the gut microbiota of the mice administered with different treated tomato juice. Histograms of LDA scores in (A) Fresh and Blank, (B) HTST and Blank, and (C) HHP and Blank (LDA score (log10) ≥ 4.0) groups. Taxa enriched in the Blank group are displayed by a red bar (negative LDA score), and taxa enriched in the Fresh, HTST, and HHP tomato juice groups are described by a green bar (positive LDA score); Figure S3: Classification of mice fecal metabolites based on (A) KEGG library and (B) HMDB library. The horizontal axis represents the KEGG compounds classification, and the vertical axis represents the number of compounds in that category. The selected HMDB classifications (each pie chart) and the percentage of metabolites (each area in a pie chart) are displayed in descending order by the number of metabolites. Table S1: Chromatographic conditions; Table S2: Serum biochemical parameters in mice administered with tomato juice; Table S3: Differential metabolites discriminated between mice administered fresh, HPP-, and HTST-processed tomato juice.

## References

[1] Balasubramaniam V.M., Martinez-Monteagudo S.I., Gupta R. (2015). Principles and application of high pressure-based technologies in the food industry. Annu. Rev. Food Sci. Technol..

[2] Shahbaz H.M., Kim J.U., Kim S.-H., Park J., Grumezescu A.M., Holban A.M. (2018). Advances in Nonthermal Processing Technologies for Enhanced Microbiological Safety and Quality of Fresh Fruit and Juice Products. Food Processing for Increased Quality and Consumption.

[3] Wang C.Y., Wang Y.T., Wu S.J., Shyu Y.T. (2018). Quality changes in high hydrostatic pressure and thermal pasteurized grapefruit juice during cold storage. J. Food Sci. Technol..

[4] Wang F., Du B.L., Cui Z.W., Xu L.P., Li C.Y. (2017). Effects of high hydrostatic pressure and thermal processing on bioactive compounds, antioxidant activity, and volatile profile of mulberry juice. Food Sci. Technol. Int..

[5] Elizondo-Montemayor L., Hernandez-Brenes C., Ramos-Parra P.A., Moreno-Sanchez D., Nieblas B., Rosas-Perez A.M., Lamadrid-Zertuche A.C. (2015). High hydrostatic pressure processing reduces the glycemic index of fresh mango puree in healthy subjects. Food Funct..

[6] Li D., Wang P., Wang P., Hu X., Chen F. (2019). Targeting the gut microbiota by dietary nutrients: A new avenue for human health. Crit. Rev. Food Sci. Nutr..

[7] Carmody R.N., Gerber G.K., Luevano J.M., Gatti D.M., Somes L., Svenson K.L., Turnbaugh P.J. (2015). Diet dominates host genotype in shaping the murine gut microbiota. Cell Host Microbe.

[8] Zmora N., Suez J., Elinav E. (2019). You are what you eat: Diet, health and the gut microbiota. Nat. Rev. Gastro. Hepat..

[9] Shanmugam H., Ganguly S., Priya B. (2022). Plant food bioactives and its effects on gut microbiota profile modulation for better brain health and functioning in Autism Spectrum Disorder individuals: A review. Food Front..

[10] Morrison D.J., Preston T. (2016). Formation of short chain fatty acids by the gut microbiota and their impact on human metabolism. Gut Microbes.

[11] Zhang Z., Li D. (2018). Thermal processing of food reduces gut microbiota diversity of the host and triggers adaptation of the microbiota: Evidence from two vertebrates. Microbiome.

[12] Perez-Burillo S., Pastoriza S., Jimenez-Hernandez N., D’Auria G., Francino M.P., Rufian-Henares J.A. (2018). Effect of food thermal processing on the composition of the gut microbiota. J. Agric. Food Chem..

[13] FAO Tomato Production. https://www.fao.org/faostat/en/#data.

[14] Salehi B., Sharifi-Rad R., Sharopov F., Namiesnik J., Roointan A., Kamle M., Kumar P., Martins N., Sharifi-Rad J. (2019). Beneficial effects and potential risks of tomato consumption for human health: An overview. Nutrition.

[15] Liso M., De Santis S., Scarano A., Verna G., Dicarlo M., Galleggiante V., Campiglia P., Mastronardi M., Lippolis A., Vacca M. (2018). A Bronze-Tomato Enriched Diet Affects the Intestinal Microbiome under Homeostatic and Inflammatory Conditions. Nutrients.

[16] Garcia-Alonso F.J., Gonzalez-Barrio R., Martin-Pozuelo G., Hidalgo N., Navarro-Gonzalez I., Masuero D., Soini E., Vrhovsek U., Periago M.J. (2017). A study of the prebiotic-like effects of tomato juice consumption in rats with diet-induced non-alcoholic fatty liver disease (NAFLD). Food Funct..

[17] Wang X., Chen F., Ma L., Liao X., Hu X. (2021). Non-volatile and volatile metabolic profiling of tomato juice processed by high-hydrostatic-pressure and high-temperature short-time. Food Chem..

[18] Ruxton C.H.S., Myers M. (2021). Fruit juices: Are they helpful or harmful? An evidence review. Nutrients.

[19] He W.S., Li L., Rui J., Li J., Sun Y., Cui D., Xu B. (2020). Tomato seed oil attenuates hyperlipidemia and modulates gut microbiota in C57BL/6J mice. Food Funct..

[20] Ley R.E., Turnbaugh P.J., Klein S., Gordon J.I. (2006). Human gut microbes associated with obesity. Nature.

[21] Krajmalnik-Brown R., Ilhan Z.E., Kang D.W., DiBaise J.K. (2012). Effects of gut microbes on nutrient absorption and energy regulation. Nutr. Clin. Pract..

[22] McKee A.M., Kirkup B.M., Madgwick M., Fowler W.J., Price C.A., Dreger S.A., Ansorge R., Makin K.A., Caim S., Le Gall G. (2021). Antibiotic-induced disturbances of the gut microbiota result in accelerated breast tumor growth. iScience.

[23] Downes J., Dewhirst F.E., Tanner A.C.R., Wade W.G. (2013). Description of *Alloprevotella rava* gen. nov., sp. nov., isolated from the human oral cavity, and reclassification of *Prevotella tannerae* Moore et al. 1994 as *Alloprevotella tannerae* gen. nov., comb. nov. Int. J. Syst. Evol. Microbiol..

[24] Eilam O., Zarecki R., Oberhardt M., Ursell L.K., Kupiec M., Knight R., Gophna U., Ruppin E. (2014). Glycan degradation (GlyDeR) analysis predicts mammalian gut microbiota abundance and host diet-specific adaptations. mBio.

[25] Liu X., Mao B., Gu J., Wu J., Cui S., Wang G., Zhao J., Zhang H., Chen W. (2021). *Blautia*-a new functional genus with potential probiotic properties?. Gut Microbes.

[26] Shang Q., Shan X., Cai C., Hao J., Li G., Yu G. (2016). Dietary fucoidan modulates the gut microbiota in mice by increasing the abundance of *Lactobacillus* and *Ruminococcaceae*. Food Funct..

[27] Eeckhaut V., Machiels K., Perrier C., Romero C., Maes S., Flahou B., Steppe M., Haesebrouck F., Sas B., Ducatelle R. (2013). *Butyricicoccus pullicaecorum* in inflammatory bowel disease. Gut.

[28] Zhang T., Li Q., Cheng L., Buch H., Zhang F. (2019). Akkermansia muciniphila is a promising probiotic. Microb. Biotechnol..

[29] McIntosh C.M., Chen L., Shaiber A., Eren A.M., Alegre M.L. (2018). Gut microbes contribute to variation in solid organ transplant outcomes in mice. Microbiome.

[30] Tong A., Wu W., Chen Z., Wen J., Jia R., Liu B., Cao H., Zhao C. (2023). Modulation of gut microbiota and lipid metabolism in rats fed high-fat diets by *Ganoderma lucidum* triterpenoids. Curr. Res. Food Sci..

[31] Liu T.H., Tao W.C., Liang Q.E., Tu W.Q., Xiao Y., Chen L.G. (2020). Gut microbiota-related evidence provides new insights into the association between activating transcription factor 4 and development of salt-induced hypertension in mice. Front Cell Dev. Biol..

[32] Dziedzic K., Gorecka D., Szwengiel A., Michniewicz J., Drozdzynska A., Walkowiak J. (2019). Interactions between fecal bacteria, bile acids and components of tomato pomace. Food Sci. Biotechnol..

[33] Wu T., Gao Y., Hao J., Yin J., Li W., Geng J., Liu R., Sui W., Zhang M. (2019). Lycopene, amaranth, and sorghum red pigments counteract obesity and modulate the gut microbiota in high-fat diet fed C57BL/6 mice. J. Funct. Foods.

[34] Johnson E.L., Heaver S.L., Waters J.L., Kim B.I., Bretin A., Goodman A.L., Gewirtz A.T., Worgall T.S., Ley R.E. (2020). Sphingolipids produced by gut bacteria enter host metabolic pathways impacting ceramide levels. Nat. Commun..

[35] Brown E.M., Ke X., Hitchcock D., Jeanfavre S., Avila-Pacheco J., Nakata T., Arthur T.D., Fornelos N., Heim C., Franzosa E.A. (2019). Bacteroides-derived sphingolipids are critical for maintaining intestinal homeostasis and symbiosis. Cell Host Microbe.

[36] Farooqui A.A., Horrocks L.A., Farooqui T. (2007). Interactions between neural membrane glycerophospholipid and sphingolipid mediators: A recipe for neural cell survival or suicide. J. Neurosci. Res..

[37] Neis E.P., Dejong C.H., Rensen S.S. (2015). The role of microbial amino acid metabolism in host metabolism. Nutrients.

[38] Nakayama Y. (2021). *Corynebacterium glutamicum* mechanosensing: From osmoregulation to L-glutamate secretion for the avian microbiota-gut-brain axis. Microorganisms.

[39] Hibberd M.C., Wu M., Rodionov D.A., Li X., Cheng J., Griffin N.W., Barratt M.J., Giannone R.J., Osterman O.A., Hettich R.L. (2017). The effects of micronutrient deficiencies on bacterial species from the human gut microbiota. Sci. Transl. Med..

[40] Gomez V.P.B.S., Martin-Pozuelo G., Santaella M., Periago M.J. (2011). Effect of tomato juice intake on lycopene and short chain fatty acid content in faeces of Sprague Dawley rats. Ann. Vet. Anim. Sci..

[41] Elizondo-Montemayor L., Ramos-Parra P.A., Jacobo-Velázquez D.A., Treviño-Saldaña N., Marín-Obispo L.M., Ibarra-Garza I.P., Garcia-Amezquita L.E., Del Follo-Martínez A., Welti-Chanes J., Hernández-Brenes C. (2020). High hydrostatic pressure stabilized micronutrients and shifted dietary fibers, from insoluble to soluble, producing a low-glycemic index mango pulp. CyTA J. Food.

[42] Holscher H.D. (2017). Dietary fiber and prebiotics and the gastrointestinal microbiota. Gut Microbes.

[43] Huang W., Fang Q., Fan L., Hong T., Tan H., Nie S. (2022). Pectin with various degrees of esterification differentially alters gut microbiota and metabolome of healthy adults. eFood.

[44] Parker B.J., Wearsch P.A., Veloo A.C.M., Rodriguez-Palacios A. (2020). The genus *Alistipes*: Gut bacteria with emerging implications to inflammation, cancer, and mental health. Front Immunol..

[45] Salem F., Kindt N., Marchesi J.R., Netter P., Lopez A., Kokten T., Danese S., Jouzeau J.Y., Peyrin-Biroulet L., Moulin D. (2019). Gut microbiome in chronic rheumatic and inflammatory bowel diseases: Similarities and differences. United Eur. Gastroent..

[46] Akhtar M., Naqvi S.U., Liu Q., Pan H., Ma Z., Kong N., Chen Y., Shi D., Kulyar M.F., Khan J.A. (2022). Short chain fatty acids (SCFAs) are the potential immunomodulatory metabolites in controlling *Staphylococcus aureus*-mediated mastitis. Nutrients.

