# Supplementary material for: Gut Microbiome and Metabolome Modulation by High-Hydrostatic-Pressure-Processed Tomato Juice
Source: Nutrients. 2024 Feb 29;16(5):710. doi: 10.3390/nu16050710 (PMC10934475; doi:10.3390/nu16050710)
Supplement: Supplementary file 1 [file nutrients-16-00710-s001.zip › Supplementary materials.pdf]

Table S1 Chromatographic conditions

Time（min）	flow rate（mL/min）	Gradient elution procedure		Curve
		Solvent A: 0.1% formic acid in water: acetonitrile (95:5, v/v)	Solvent B: 0.1% formic acid in acetonitrile: isopropanol: water (47.5:47.5:5, v/v)	
0	0.40	100%	0%	6
3.5	0.40	75.5%	24.5%	6
5	0.4	35%	65%	6
5.5	0.4	0%	100%	6
7.4	0.6	0%	100%	6
7.6	0.6	48.5	51.5%	6
7.8	0.5	100%	0%	6
9	0.4	100%	0%	6
10	0.4	100%	0%	6

Table S2 Serum biochemical parameters in mice administered with tomato juice

	HTST	HHP	Fresh	Blank
TG（mmol/L）	0.90 ± 0.15[a]	1.01 ± 0.23[a]	0.95 ± 0.13[a]	1.01 ± 0.13[a]
TC（mmol/L）	2.60 ± 0.35[a]	2.55 ± 0.24[a]	2.69 ± 0.41[a]	2.69 ± 0.20[a]
HDL-C（mmol/L）	1.69 ± 0.20[a]	1.68 ± 0.23[a]	1.71 ± 0.37[a]	1.76 ± 0.16[a]
LDL-C（mmol/L）	0.28 ± 0.04[a]	0.30 ± 0.02[a]	0.31 ± 0.04[a]	0.28 ± 0.04[a]
ALT（U/L）	161.50 ± 29.12[a]	156.78 ± 23.89[a]	149.35 ± 22.81[a]	164.12 ± 30.13[a]

TG, total triacylglycerol; TC, total cholesterol; HDL-C, high-density lipoprotein cholesterol; LDL-C, low-density lipoprotein cholesterol; Mean ± SEM (n = 12). Different letters in the same line indicate significant differences ($p < 0.05$). Blank: normal saline control group; Fresh: fresh tomato juice administration group; HHP: high hydrostatic pressure processed tomato juice administration group; HTST: high-temperature short-time processed tomato juice administration group.

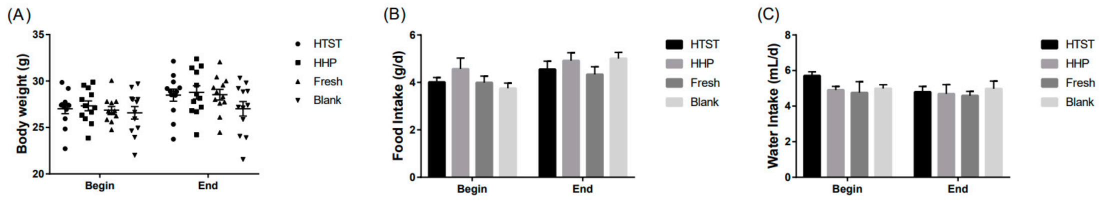

Figure S1. (A)The body weight, (B)food consumption, and (C)water intake of mice administered with tomato juice.

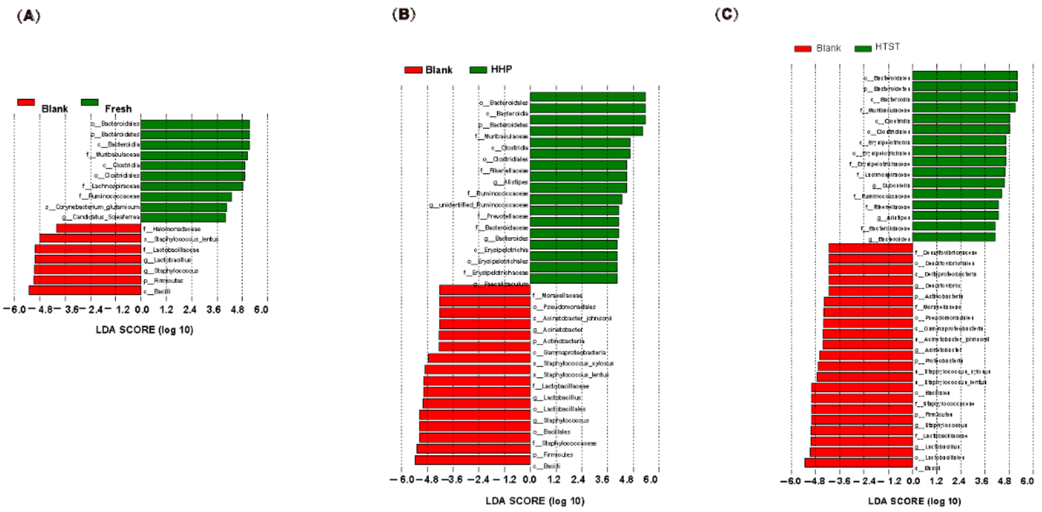

Figure S2 Linear discriminant analysis effect size analysis to identify taxonomic differences in the gut microbiota of the mice administrated with different treated tomato juice. Histograms of LDA scores in (A) Fresh and Blank, (B) HTST and Blank, and (C) HHP and Blank (LDA score (log10) ≥ 4.0) group. Taxa enriched in the Blank group are displayed by red bar (negative LDA score), and taxa enriched in the Fresh, HTST, and HHP tomato juice groups are described by green bar (positive LDA score).

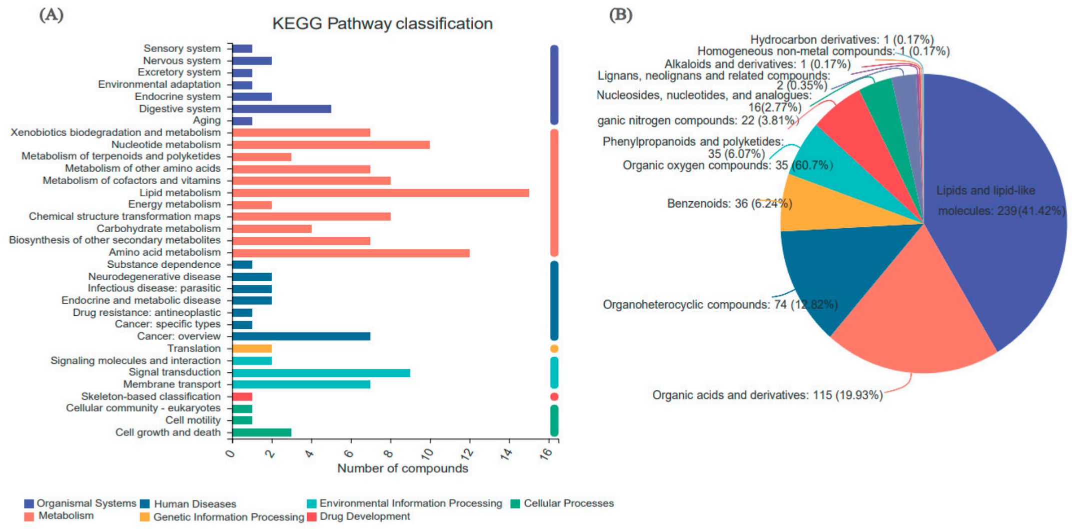

Figure S3 Classification of mice fecal metabolites based on (A) KEGG library and (B) HMDB library. The horizontal axis represents the KEGG compounds classification, and the vertical axis represents the number of compounds in that category. The selected HMDB classifications (each pie chart) and the percentage of metabolites (each area in a pie chart) are displayed in descending order by the number of metabolites.
